# Rapid Conversion of a Biomedical Engineering Laboratory from in Person to Online

**DOI:** 10.1007/s43683-020-00031-y

**Published:** 2020-09-25

**Authors:** H. Lancashire, A. Vanhoestenberghe

**Affiliations:** 1 Department of Medical Physics and Biomedical Engineering, University College London, London, UK; 2 Aspire Centre for Rehabilitation Engineering and Assistive Technology, University College London, London, UK

**Keywords:** Biomedical engineering, Online, Rapid delivery, Intended learning outcomes, Laboratory teaching

## Abstract

Temporary higher education institution closures in response to the 2020 COVID-19 pandemic disrupted student teaching. This paper reports on the rapid conversion of an in person laboratory session to online delivery, within 24 h of the previously scheduled in person session, and two working days after the end of face-to-face teaching at the authors’ institution. To ensure teaching continuity for students, and address intended learning outcomes (ILOs) where possible, we created online material rapidly in a manner familiar to students. Online material followed the same structure as a previously released laboratory script, intended for the in person session, and was presented on the institutional Virtual Learning Environment. The online material comprised experimental data in tables and equipment readouts, brief descriptions, and short videos demonstrating the experimental methods. We assess to what extent the ILOs were met, and argue that clear ILOs help guide changes to teaching methods, to reduce any disruption to student learning. Four aspects of the initiative are highlighted: rapid delivery; familiar structure; familiar delivery; and videos used for emphasis.

## Challenge Statement

Laboratory and field work are essential parts of professional science and engineering practice, and therefore must be part of the higher education curriculum.^2^,^8^ Laboratory based teaching has benefits beyond the hands on practice of experimentation, helping students gain skills of scientific argument and inquiry.^6^ In person laboratory based teaching is among the most impacted during any disruption to education. This is due to the relatively high costs, time intensiveness, and staffing requirements of in person laboratory teaching.^4^,^8^

**Figure 1:**
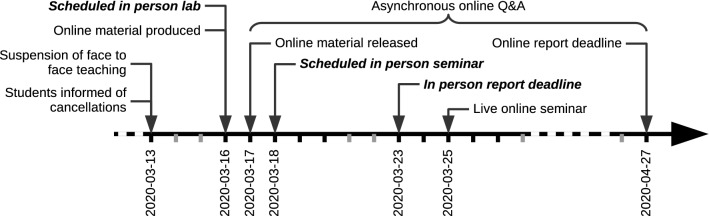
Detailed timeline of events. *Bold italics* indicate cancelled activities. Grey ticks indicate weekends.

This paper reports on the challenge of rapidly converting scheduled in person laboratory teaching to online teaching in response to the COVID-19 pandemic. The in person laboratory session was scheduled to take place on the first teaching day after the suspension of face-to-face teaching at the authors’ institution, therefore rapid conversion was essential to minimise disruption to students. Key intended learning outcomes (ILOs) for the course were presented in the laboratory session; therefore it was essential that this material was provided to students. This initiative aimed to meet this challenge while providing teaching continuity for students and retain the intended learning where possible. A detailed timeline is given in Fig. 1.

## The Biomedical Engineering Teaching Context

Within the Integrated Engineering Programme (IEP) at University College London, undergraduate degrees in Biomedical Engineering are offered by the Department of Medical Physics and Biomedical Engineering.^9^ Key elements of the IEP curriculum include practical engineering, an alignment to research, and problem based learning. This paper presents work done for a laboratory session of the module Medical Electronics and Neural Engineering (hereafter “the module”) which comprises 150 study hours including lectures, seminars, laboratory sessions, and self directed learning. The module is also available as an elective for students with an appropriate background at undergraduate and postgraduate levels. In the 2019/20 academic year, 24 students studied the module.

The module includes three compulsory laboratory assignments each comprising 4 to 8 h of practical experimentation followed by a written report. The laboratory assignments aim to develop fundamental understanding of the application of electronics in medicine, teach pragmatism and problem solving skills and demonstrate key physiological, chemical, and electrical concepts. Each session is taught by two or three staff members (demonstrators), including the authors and postgraduate teaching assistants.

## The In Person Muscle Stimulator Laboratory

During the in person laboratory session, students characterise a muscle stimulator and then use this to investigate the physiological response to electrical stimulation. The ILOs are given in Table 1. Students work in groups of two or three, following a laboratory script which guides them through practical aspects of the assignment, and prompts with appropriate discussion questions.

**Table 1:** The general (GILOs) and practical intended learning outcomes (PILOs).

General ILOs	**GILO1:** How a transformer in a stimulator output stage can distort the output waveform.	**Fully met.** Oscilloscope screenshots of distorted stimulator outputs were given alongside video of the transformer response to increasing amplitude stimulation. Students interpreted these results in their reports
	**GILO2:** Two reasons why, despite these distortions, transformers are common in stimulator output stages.	**Fully met.** Students were expected to use prior knowledge and presented results to arrive at the expected reasons and present this in their reports
	**GILO3:** Several aspects of the physiological response to electrical stimulation, including the effect of the pulse width and charge delivered on the motor threshold (strength-duration curve), and the effect of the frequency.	**Partially met.** As part of the written assignment students were expected to calculate and present a strength duration curve. The effect of frequency was presented in a video; however, as with PILO4 students could not experience this directly
Practical ILOs	**PILO1:** Some more practice with oscilloscope, in particular how to use the external trigger mode.	**Not met.** In two previous in-person laboratory sessions, students gained experience with oscilloscopes. The use of the external trigger was demonstrated on video, but students could not attempt this themselves
	**PILO2:** How to use a capacitor to measure the charge in a pulse.	**Fully met.** Oscilloscope screenshots of the capacitor response were given and students were expected to compare calculated charge using both resistor and capacitor methods, and to interpret these results in their written assignment
	**PILO3:** How to record an experimental strength-duration curve and derive the rheobase and chronaxie.	**Partially met.** As part of the written assignment students were expected to derive rheobase and chronaxie from the data provided. As data for an appropriate range of pulse width was given, the students could not experiment with the pulse width values and observe whether their choice was appropriate
	**PILO4:** What electrical stimulation feels like, for different current amplitudes and frequencies.	**Not met.** Students were unable to experience electrical stimulation themselves. To partially address this, sensations were described, live, in videos provided as part of the online material

The in person laboratory session is designed to meet all ILOs fully, either through practice or in the students’ written laboratory report. We have reflected on the extent to which the ILOs were met with the online material

During the session demonstrators are available to answer student questions at any stage during the practical session. Typical student questions include: advice on the use of equipment; clarification of the laboratory script; explanations of the phenomena observed. Students are encouraged to discuss among themselves and try to solve queries before asking for assistance, and demonstrators guide students towards solutions through enquiry rather than by providing immediate complete answers.

Characterisation includes the use of an oscilloscope to observe the stimulus timing and the current controlled output stage. Students observe the charge recovery of the stimulator using a model cell connected to the stimulator output. Finally students carry out an investigation of the physiological effects of muscle stimulation, including recording a strength-duration curve.^3^ Clear safety warnings and instructions are given about the safe use of a stimulator and students are allowed to opt out of receiving muscle stimulation at any time without penalty. The motor threshold current amplitudes of a non-dominant arm biceps muscle are observed at a range of pulse widths chosen by the students, and students use these to plot a strength-duration curve and calculate values for rheobase and chronaxie. Students then investigate the effect of the stimulation frequency on the motor response, including tetanic contraction and the sensation of electrical stimulation.

Students submit a short individual laboratory report which assesses both their experimental ability and their understanding of the concepts. The report covers: practical results, such as an appropriate oscilloscope screenshots; values calculated from experiments; and open ended enquiry considering safety, limitations, comparisons, and explanations. Reports are submitted online though the institutional virtual learning environment (VLE, Moodle), and are automatically anonymised for marking.

## Novel Initiative

Our approach to online teaching aimed to minimise disruption to students. Therefore, material was produced rapidly, followed the script provided to students prior to the laboratory session, and was presented using familiar tools.

**Figure 2:**
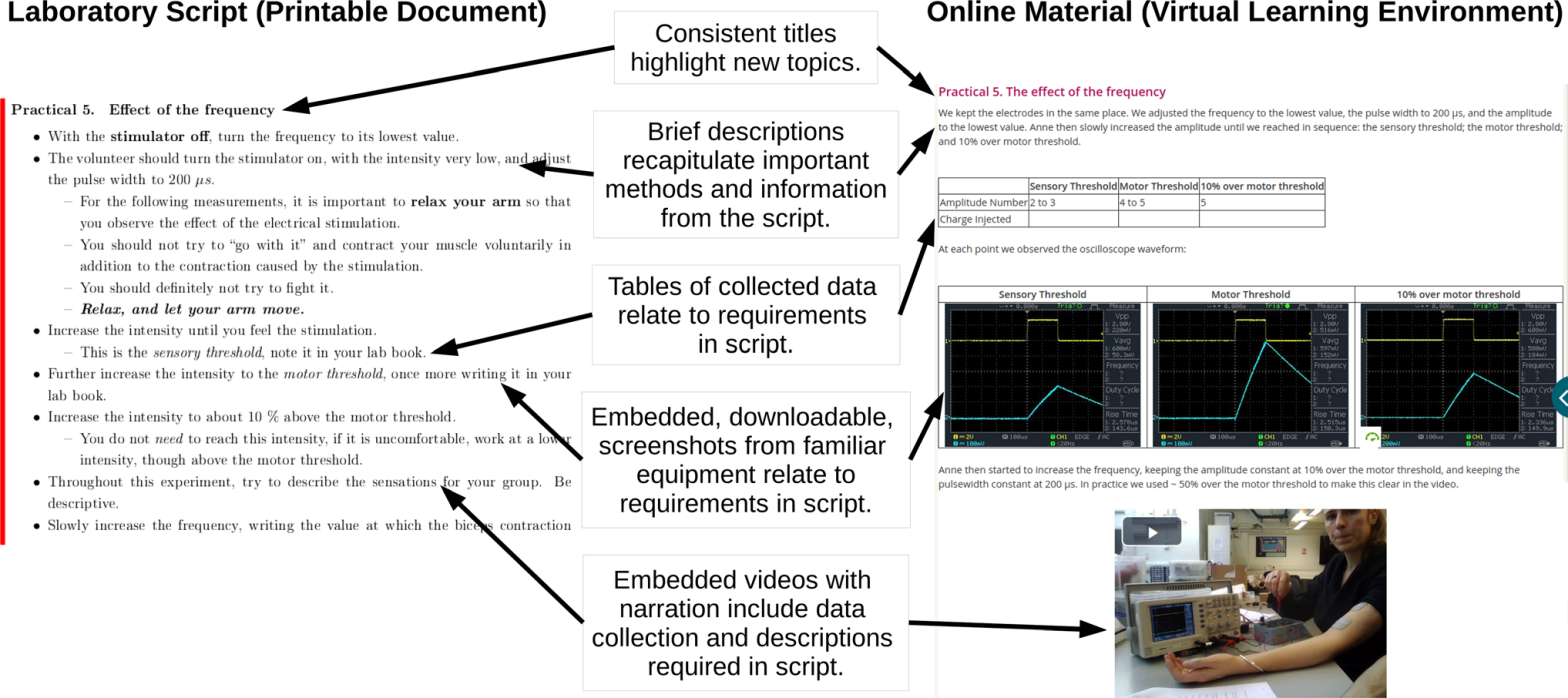
The online material (right) was presented in a manner consistent with the provided laboratory script (left). Students who had prepared for the in-person laboratory by reading the script would expect material organised this way.

To prepare the online material we worked through the laboratory practical. Each practical step reported on the VLE was titled according to the convention in the script, and had a brief introductory paragraph describing the experimental setup (see Fig. 2). Short videos of each practical step were recorded using an embedded system in the VLE to ensure compatibility. This enabled us to embed videos of up to 2 min in length directly alongside other material on the VLE. Videos were arranged to ensure students could see the equipment in use, the oscilloscope display, and the arm being stimulated.^10^ Videos included audio narration explaining key points such as the experimental setup, what data was being collected, and the sensation of electrical stimulation, to keep students connected with the practical process.^2^,^8^,^10^ Experimental data was recorded primarily as oscilloscope screenshots, see Fig. 2. This ensured that students could practice reading data from an oscilloscope display, and could clearly see the waveforms observed. This approach was already used by most students during in person laboratories, therefore a similar level of abstraction was maintained.^8^ Where experimental data could not be displayed on the oscilloscope (for example stimulation amplitude in arbitrary units), this was recorded in tables of data on the VLE. All online material was developed by two members of the teaching team, in the course of a 3 h session in the laboratory the day before building closure.

The material was released to students after one round of internal review, including error checking, and ensuring that the videos were playable on alternative platforms. The material was released within 24 h of the scheduled in person session (see Fig. 1). A seminar focussed on the ILOs of all the laboratory practicals was delayed by 1 week to allow students to familiarise themselves with the online material, and was carried out live online using the VLE. In parallel with the live seminar, and as is already our common practice during normal operations, students asked questions using an anonymous forum on the VLE, answers were asynchronous, and students are encouraged to respond to each others’ questions to support collaborative working. We confirmed that all the material required for the laboratory report was available online and therefore did not make any changes to the required submission except for extending the submission deadline following institutional guidelines, to reduce negative impacts on students due to the ongoing disruption.

## Reflection

With this initiative we had a key aim: to address the intended learning outcomes where possible. Two critical parts of our approach contributed to meeting this aim: by rapidly adapting in person material to online; and by providing continued support for student learning. The approach allowed us to ensure teaching continuity while minimising undue challenge and disruption to students.

Firstly, the assignment was rapidly converted to online delivery, with 24 h delay between the intended practical date and the online material release date. By providing the assignment to students rapidly and extending the submission deadline we intended to minimise changes to students’ planned work schedules and allow them to manage their workload more easily. Our approach minimised the additional workload on the teaching staff, using facilities we as a teaching team were already familiar with, in time already allocated to teaching, ensuring rapid delivery.

Second, the assignment followed the script provided to students prior to the laboratory session. Students were asked to prepare for the in person session, and we intended that this preparation would be equally useful for the online assignment with clear, consistent sections.^10^

Third, the online material was delivered using systems students were familiar with (Moodle), within minimal complexity: the online material was analogous to a complete student lab book following the in person practical session. The VLE enabled students to access the resources at any time, regardless of location, to learn at their own pace,^5^ although we have anecdotal reports that some students outside the UK require a virtual private network (VPN) to access our institution’s VLE placing either costs or internet bandwidth restrictions on students. We concurred that delivering materials using alternative tools, for example requiring students to navigate away from the assignment page to view videos or tables of results, would increase staff and student workloads without meeting the ILOs to a greater extent.

Fourth, videos were used for emphasis, to connect students with the practical experiments.^8^ Videos were short, presenting only one practical step, this is preferred by students and improves viewer retention.^10^ We avoided providing the key information in video form only, to keep this material accessible for all students, and so that the material could be navigated easily.

Table 1 shows to what extent each ILO was met, as determined by the teaching team. Having clear ILOs split into general and practical ILOs, which has been our practice for many years, was key to choosing an appropriate delivery method. We could rapidly evaluate which ILOs would be met by which delivery methods to ensure that the essential learning was delivered: conceptual understanding ILOs were more easily met than practical or design learning.^1^,^7^,^8^ General ILOs were assessed though laboratory reports, where questions either directly addressed the concept, or required its use to arrive at an answer. In contrast practical skills could not be taught or assessed using the present approach and we identified these prior to the teaching (Table 1). Where it was not possible to meet the ILOs with online teaching we addressed this directly as part of the online material, either highlighting where this was covered during previous sessions, or clarifying to students that the ILO would not be met. We argue that ensuring all teaching material has clear ILOs will help with the evaluation of any future rapid change to teaching methods, and will help reduce the impact on student learning when teaching disruption occurs.

Students commented that the lab was clearly laid out, but not being able to interact with the equipment and not being able to ask live questions were clear limitations. This supports our approach of keeping the online material consistent with the provided laboratory script and using systems familiar to students, and reiterates the limitations to the ILOs we identified with the online approach. Similar to the in person assignments students submitted individual laboratory reports following the online laboratory. For the online assignment students were not restricted from working in groups in a similar manner to the in person sessions, with whole group discussion using the anonymous forum encouraged. It was however not possible to assess individual students’ engagement online, whereas in person teaching staff work to include all students in discussions.

We propose to evaluate this initiative by comparing student results with the in person laboratory teaching delivered in 2018/19, accounting for student attainment in laboratory assignments delivered in person to both student cohorts. The outcomes of this comparison are restricted to internal use within our organisation for the purposes of teaching evaluation only.

Whilst we make no claim that this initiative demonstrates best practice online teaching, this approach met our aims rapidly. This approach would scale for an entire laboratory course of 40 + contact hours, maintaining scope for scientific argument and analysis. However, the lack of hands-on science, and the directed nature of the online material, removes much of the inquiry present in the laboratory, and means we can only recommend this approach for short term rapidly converted teaching.^6^ Further, this approach is only appropriate where access to laboratories is maintained for teaching staff. We are currently planning connected online teaching for the academic year 2020/21, by combining video demonstrations with an experimental simulation to generate student specific results.^5^ With this approach we expect to better meet the ILOs, and increase student engagement with scientific inquiry^6^; however any distance learning approach will not enable students to experience the sensation of muscle electrical stimulation.

## Data Availability

The teaching material is available on reasonable request to the corresponding author.

## References

[1] Bourne J, Harris D, Mayadas F (2005). Online engineering education: learning anywhere, anytime. J Eng Educ..

[2] Fung D (2017). A connected curriculum for higher education.

[3] Geddes LA, Bourland JD (1985). The strength-duration curve. IEEE Trans Biomed Eng..

[4] Gomes L, Bogosyan S (2009). Current trends in remote laboratories. IEEE Trans Ind Electron..

[5] Heradio R, de la Torre L, Galan D, Cabrerizo FJ, Herrera-Viedma E, Dormido S (2016). Virtual and remote labs in education: a bibliometric analysis. Comput Educ..

[6] Hofstein A, Taber KS, Akpan B (2017). The role of laboratory in science teaching and learning. Science education.

[7] Lindsay ED, Good MC (2005). Effects of laboratory access modes upon learning outcomes. IEEE Trans Educ..

[8] Ma J, Nickerson JV (2006). Hands-on, simulated, and remote laboratories: a comparative literature review. ACM Comput Surv..

[9] Mitchell JE, Nyamapfene A, Roach K, Tilley E (2019). Faculty wide curriculum reform: the integrated engineering programme. Eur J Eng Educ..

[10] Rodgers TL, Cheema N, Vasanth S, Jamshed A, Alfutimie A, Scully PJ (2020). Developing pre-laboratory videos for enhancing student preparedness. Eur J Eng Educ..

